# Estimating the individual stillborn rate from easy-to-collect sow data on farm: an application of the bayesian network model

**DOI:** 10.1186/s40813-024-00395-5

**Published:** 2024-10-16

**Authors:** Charlotte Teixeira Costa, Gwenaël Boulbria, Christophe Dutertre, Céline Chevance, Théo Nicolazo, Valérie Normand, Justine Jeusselin, Arnaud Lebret

**Affiliations:** 1 Rezoolution, ZA de Gohélève, Noyal-Pontivy, 56920 France; 2 HumanoIA, 338 route de Philondenx, Cabidos, 64410 France

**Keywords:** Bayesian networks, Sow, Farrowing, Stillborn piglets, Predictive model, Decision-making tool

## Abstract

**Background:**

A high number of stillborn piglets has a negative impact on production and animal welfare. It is an important contributor to piglet mortality around farrowing and continues to rise with the increase of prolificacy. The objective of this study was to build a predictive model of the stillborn rate.

**Results:**

This study was performed on two farrow-to-finish farms and one farrow-to-wean farm located in Brittany, France. At each farm, the number of total born (TB), born alive (BA), stillborn piglets (S), the same data at the previous farrowing (TB_*n*− 1_, BA_*n*− 1_ and S_*n*− 1_), backfat thickness just before farrowing and at previous weaning and parity rank were recorded in our dataset of 3686 farrowings. Bayesian networks were used as an integrated modelling approach to investigate risk factors associated with stillbirth using BayesiaLab^®^ software. Our results suggest the validity of a hybrid model to predict the percentage of stillborn piglets. Three significant risk factors were identified by the model: parity rank (percentage of total mutual information: MI = 64%), S_*n*− 1_ (MI = 25%) and TB_*n*− 1_ (MI = 11%). Additionally, backfat thickness just before farrowing was also identified for sows of parity five or more (MI = 0.4%). In practice, under optimal conditions (i.e., low parity rank, less than 8% of stillborn piglets, and a prolificacy lower than 14 piglets at the previous farrowing), our model predicted a stillborn rate almost halved, from 6.5% (mean risk of our dataset) to 3.5% for a sow at the next farrowing. In contrast, in older sows with a backfat thickness less than 15 mm, more than 15% of stillborn and a prolificacy greater than 18 piglets at the previous farrowing, the risk is multiplied by 2.5 from 6.5 to 15.7%.

**Conclusion:**

Our results highlight the impact of parity, previous prolificacy and stillborn rate on the probability of stillborn. Moreover, the importance of backfat thickness, especially in old sows, must be considered. This information can help farmers classify and manage sows according to their risk of giving birth to stillborn piglets.

## Background

Preweaning mortality is a multifactorial concern with an impact on productivity and animal welfare. Stillbirth and crushing are the two main causes of piglet death before weaning [1]. While the stillborn rate varies between countries, it continues to rise with the increase of sow prolificacy on breeding farms [2, 3]. In a recent study, reported stillborn rates varied between 7.2 and 9.5% in six different European countries [4]. In another study, the stillborn rate reached, on average, 15.4% [5]. The literature contains extensive reports on factors that influence stillbirth. First, farrowing characteristics such as litter size, placental weight, location of the foetus in the uterus, duration of farrowing, birth interval, stress and human assistance can affect stillborn rates [6–10]. Additionally, it has been demonstrated that a sow with a backfat thickness (BFT) before farrowing greater than 21 mm has an increased risk of dystocia, leading to a higher stillbirth rate [11, 12]. Sows that are thin at the time of farrowing, also showed a high level of stillbirth together with a tendency to have a lower prolificacy and earlier culling [13, 14]. A farrowing duration of more than 300 min induces stress and consequently tends to increase the stillborn rate [15]. Moreover, non-infectious factors such as the genetic type of the sow, parity rank, nutritional deficiencies and housing conditions should be considered [16–20]. Non-infectious effects on piglets (weight, sex and vitality at birth) should also be taken into account [21, 22]. Finally, infectious factors, such as porcine respiratory and reproductive syndrome (PRRS), infection with pathogenic *Leptospira*, porcine circovirus type 2, and swine influenza, should be investigated and managed. In parallel, it should be borne in mind that preweaning mortality increases with the stillborn rate [23, 24].

Several control measures can be suggested based on these recent findings to reduce the risk of stillbirth, thereby reducing preweaning mortality on farms. However, the effective implementation of these upstream interventions relies on farmer involvement, primarily by monitoring at-risk sows, but also by improving conditions for successful farrowing, i.e. less stress and an optimal environment [25]. Bayesian networks constructed using machine-learning algorithms provide a potential approach to the above-mentioned challenges to characterize the risk of stillbirth for each sow. Instead of relying on a mechanistic description of these risk factors, Bayesian networks can estimate the percentage of stillborn by describing conditional dependencies among variables in observational datasets.

To the best of our knowledge, no study on stillbirth has developed a predictive model to identify at-risk sows since the one proposed by T.E. Blackwell in 1987 [26]. This old grid aimed to determine the risk of stillbirth in sows according to their parity and previous farrowing characteristics. In this context, the aim of our study was to construct a predictive model of the stillborn rate using Bayesian networks, with the goal of providing practical tools for decision-making on the implementation of preventive measures.

## Materials and methods

### Included farms and recorded data

This study was performed on two farrow-to-finish farms and one farrow-to-wean farm located in Brittany, France. The farm characteristics and vaccination protocols in place in livestock are described in Table 1.

**Table 1:** Farms^1^ characteristics and sow vaccination protocols

	Farm 1	Farm 2	Farm 3
Farm management	farrow-to-finish	farrow-to-finish	farrow-to-wean
Number of sows	1000	600	600
Batch management	10 batches/2 weeks	10 batches/2 weeks	20 batches/week
Age at weaning ± SD (days)	23 ± 1.4	23 ± 1.4	23 ± 1.4
Maternal genetic type	F. LW x F. LD	F. LW x F. LD	F. LW x F. LD x TZ
Paternal genetic type	Pietrain	Pietrain	Pietrain
Sow vaccinations	PRRS MLV, parvovirus, swine erysipelas, *Escherichia coli*	PRRS MLV, parvovirus, swine erysipelas, *Escherichia coli* and *Clostridium perfringens* type C	PRRS MLV, parvovirus, swine erysipelas, *Escherichia coli* and *Clostridium perfringens* type C

^1^Farms randomly coded as 1,2, or 3 due to anonymity requirements. F. LW: French Large White, F. LD: French Landrace, TZ: Tai Zumu, PRRS MLV: Porcine Respiratory and Reproductive Syndrome Modified Live Virus, SD: Standard Deviation

During the study period, the three farms were regularly monitored for PRRS stability according to the American Association of Swine Veterinarians classification procedure [27]. The farms were also regularly visited by veterinary practitioners and no clinical signs of porcine diseases that should be included in the differential diagnosis of stillbirth were observed. On each farm, nine explanatory variables were recorded: namely, the parity rank, the number of total born (TB), the number born alive (BA), the number of stillborn piglets (S) and the same data at the previous farrowing (TB_*n*− 1_, BA_*n*− 1_ and S_*n*− 1_). The backfat thickness (BFT) at the previous weaning and just before farrowing were also recorded. The backfat thickness was calculated according to the average of measurements performed on the left and right sides of the sows at the P2 position (Renco Lean-Meater^®^, Minneapolis, USA).

### Calculation of stillborn rates and variable discretisation

In practice, it is easier to use the percentages of stillborn rather than the raw data. For this purpose, the stillborn rates at farrowing (%S) and at the previous farrowing (%S_*n*−1_) were calculated as follows:

$$\:\%S=\frac{S}{TB}\:\times\:100$$

$$\:\%S\text{n-1}=\frac{S\text{n-1}}{TB\text{n-1}}\:\times\:100$$

Additionally, several categories were created to improve the readability of the results. Regarding the parity, three categories were defined: gilts and sows of parity two together, parities three and four together and older sows (parities 5 and more). This categorization was based on field practices and allowed us to streamline the analysis while retaining sufficient differentiation to identify risk factors effectively. Different categories were also created for the variables TB_*n*−1_, %S_*n*−1_, and BFT before farrowing following the automated thresholds development by the software (Table 2). Indeed, during model training, the software identified and established additional thresholds (e.g., 15% for high-risk stillborn rates) which were not initially set by the researchers. These thresholds were based on the underlying data distribution and the model’s learning process. Simulations and sensitivity analyses were conducted to validate these results, ensuring that the thresholds were robust and meaningful within the context of the dataset.

**Table 2:** Discretisation of variables used in the model

Variables measured	Categories used
Parity rank	= 0 and 1 (Gilts and parity 2) = 2 and 3 (Parities 3 and 4) = 4 and more (Parities 5 and more)
TB_*n*−1_	TB_*n*−1_ ≤ 14 piglets 14 < TB_*n*−1_ ≤ 18 piglets TB_*n*−1_ > 18 piglets
%S_*n*−1_	%S_*n*−1_ ≤ 8% 8% < %S_*n*−1_ ≤ 15% %S_*n*−1_ > 15%
BFT before farrowing	BFT ≤ 15 mm BFT > 15 mm

TB_*n*−1_: number of total born at previous farrowing, %S_*n*−1_: stillborn rates at previous farrowing, BFT: backfat thickness.

### Definitions and statistical analysis

The quantitative data were recorded in an Excel database. The average values (± standard deviation) of each variable were calculated for each selected farm. In addition, a Bayesian analysis framework was applied to predict the stillborn rate using BayesiaLab^®^ software (Bayesia S.A.S., Bayesia USA, LLC, and Bayesia Singapore Pte). Bayesian networks have been developed as prediction and decision aids in a wide variety of applications. A Bayesian network is a graphical representation of the relationships among variables. In this study, the impacts of ten explanatory variables (including the farm effect) on the stillborn rate were analysed. The model included two components [28]:

1) A directed acyclic graph in which variables are represented as nodes and relationships among them as directed edges (one-way arrows).

2) A set of conditional probability distributions for each included variable representing its statistical dependencies on other variables in the network.

Bayesian networks present several advantages over traditional statistical modelling methods, including their ability to handle nonlinear relationships among variables and to include multiple, potentially highly correlated predictor variables in one model [29].

When applied to our dataset, likelihood analysis was carried out to evaluate the prediction ability of the Bayesian network model. Owing to the artificial intelligence of the software, the ability of the Bayesian network model to predict the impacts of nine individual parameters and the farm using the evidence in the stillborn rate node was evaluated. Moreover, a sensitivity analysis was carried out to measure the sensitivity of our target node (stillbirth as a percentage) in the posterior distribution to variations in the evidence entered in other nodes of the network. The evidence sensitivity was measured as the mutual information, I(X, Y), which represents the effect of one variable (X) on another variable (Y) and is calculated as follows:

I(X, Y) = H(Y) – H(X|Y).

where H(Y) is entropy, which is the measure of the uncertainty or randomness of a variable (Y) represented by a probability distribution [30, 31].

### Model validation and testing

To validate the final model, a 10-fold cross-validation was used in supervised learning. In each iteration, nine different training sets were used for training the model, while the remaining subsample was used for validation. In this study, the Markov feature was chosen as the learning algorithm. This process was repeated ten times, ensuring that each subsample served as a validation set once. The final model performance metrics were obtained by averaging the results across all ten iterations.

## Results

### Data characteristics

In total, 3686 farrowing data events were recorded. All the descriptive results from the dataset are summarized in the Table 3. Our dataset included 43% of gilts and sows of parity 2, 24% of parities 3 and 4, and 33% of sows with parities 5 or more.

**Table 3:** Description of the dataset for the three selected farms^1^ according to the parity rank of sows (mean ± standard deviation)

Data outputs/ Parity rank	Farm 1			Farm 2			Farm 3		
	1–2	3–4	5 +	1–2	3–4	5 +	1–2	3–4	5 +
Number of farrowings	1376	678	1037	116	118	90	103	83	85
**Previous farrowing (n-1)**									
Total born/litter (TB_*n*−1_)	14.12 ± 2.8	14.55 ± 3.4	15.70 ± 3.2	15.93 ± 3.8	17.18 ± 3.2	17.83 ± 3.6	14.74 ± 2.4	15.43 ± 3.1	16.24 ± 3.8
% stillborn piglets (%S_*n*−1_)	4.58 ± 7.5	3.89 ± 6.4	7.88 ± 7.9	5.86 ± 14.8	4.11 ± 6.9	3.76 ± 5.2	5.14 ± 13.2	4.56 ± 7.2	5.69 ± 6.9
BFT at weaning, mm	12.76 ± 2.6	13.13 ± 2.8	13.69 ± 2.7	14.42 ± 3.3	15.20 ± 2.9	15.71 ± 3.3	11.68 ± 2.2	12.45 ± 2.0	12.60 ± 1.9
**At farrowing (n)**									
Total born/litter (TB)	14.08 ± 3.0	15.29 ± 3.5	15.60 ± 3.4	16.12 ± 3.6	18.03 ± 3.3	17.66 ± 3.3	14.83 ± 3.1	15.84 ± 3.6	16.28 ± 3.5
Piglets born alive/litter	13.46 ± 3.0	14.22 ± 3.3	13.96 ± 3.1	15.47 ± 3.6	17.23 ± 3.0	16.64 ± 3.4	14.25 ± 2.9	15.01 ± 3.3	14.92 ± 3.0
% stillborn piglets	4.19 ± 7.3	6.50 ± 9.0	9.97 ± 10.1	4.01 ± 7.8	4.21 ± 5.4	5.55 ± 9.0	4.63 ± 10.0	4.72 ± 7.0	7.61 ± 8.1
BFT before farrowing, mm	16.33 ± 3.1	16.59 ± 2.9	16.97 ± 3.1	17.77 ± 3.2	18.47 ± 3.6	19.00 ± 4.3	14.34 ± 2.3	15.84 ± 2.3	15.69 ± 2.7

^1^Farms randomly coded as 1,2, or 3 due to anonymity requirements. BFT: Backfat thickness

Considering the entire dataset, the average stillborn rate was 6.5% (corresponding to the average risk).

### Final model

Ultimately, Bayesian networks, as an integrated modeling approach, produced a model with an average calibration accuracy of 92%, representing the model’s probability distribution based on our dataset. Additionally, the predictability based solely on our validation dataset was 72%, with a standard deviation of 2.09%. This model included only three significant risk factors for predicting the stillbirth rate. These factors included the stillbirth rate and the number of total born at the previous farrowing and the parity rank of the sow. A fourth factor, but only for sows with 5 or more parities, namely backfat thickness just before farrowing was also included in the final model.

**Fig. 1:**
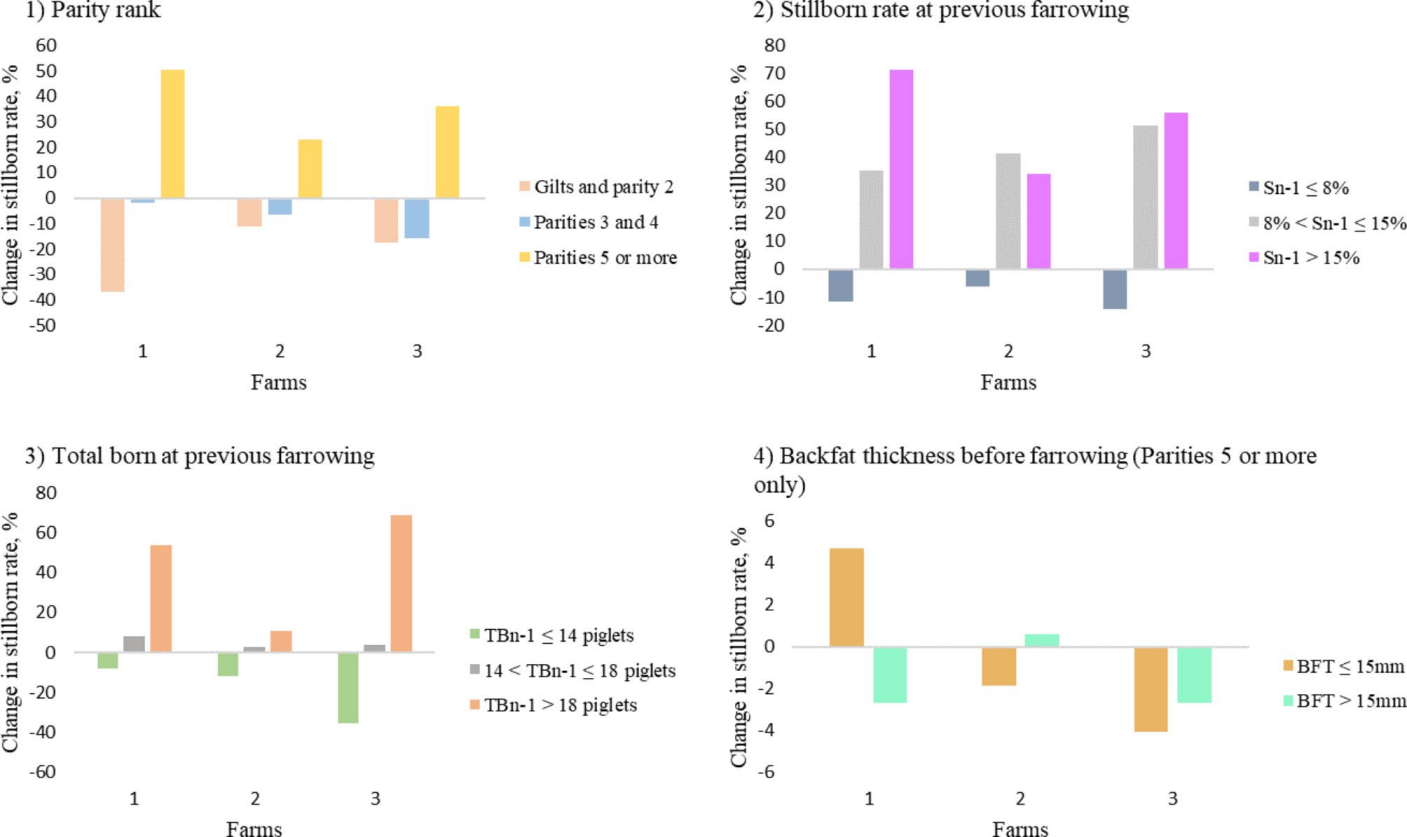
Effect of variables on the expected change in the stillborn rate compared to the baseline (fixed at 6.5% from our dataset) rate for the three farms included in the study

The impacts of each significant risk factor, taken independently, on the change of stillborn percentages are presented in Fig. 1:

In the three farms:

- The percentages of stillborn rates were lower than the baseline (6.5% in our dataset) for a gilt or parity 2 sow and higher for parities 5 or more (Fig. 1.1). For example, in the farm 1, the percentage of stillborn was 50% higher for parities 5 and more compared to the baseline.
- The percentages of stillborn rates were lower than the baseline when the stillborn rate at previous farrowing was less than or equal to 8% and higher when it exceeded 8% (Fig. 1.2).
- Also, the percentages of stillborn rates were lower than the baseline when the number of total born was less than or equal to 14 piglets at previous farrowing and higher over 18 total born (Fig. 1.3).

In the farm 1, regarding the impact of BFT of parities 5 and more, the percentages of stillborn rates were higher when BFT was less than 15 mm (Fig. 1.4).

**Fig. 2:**
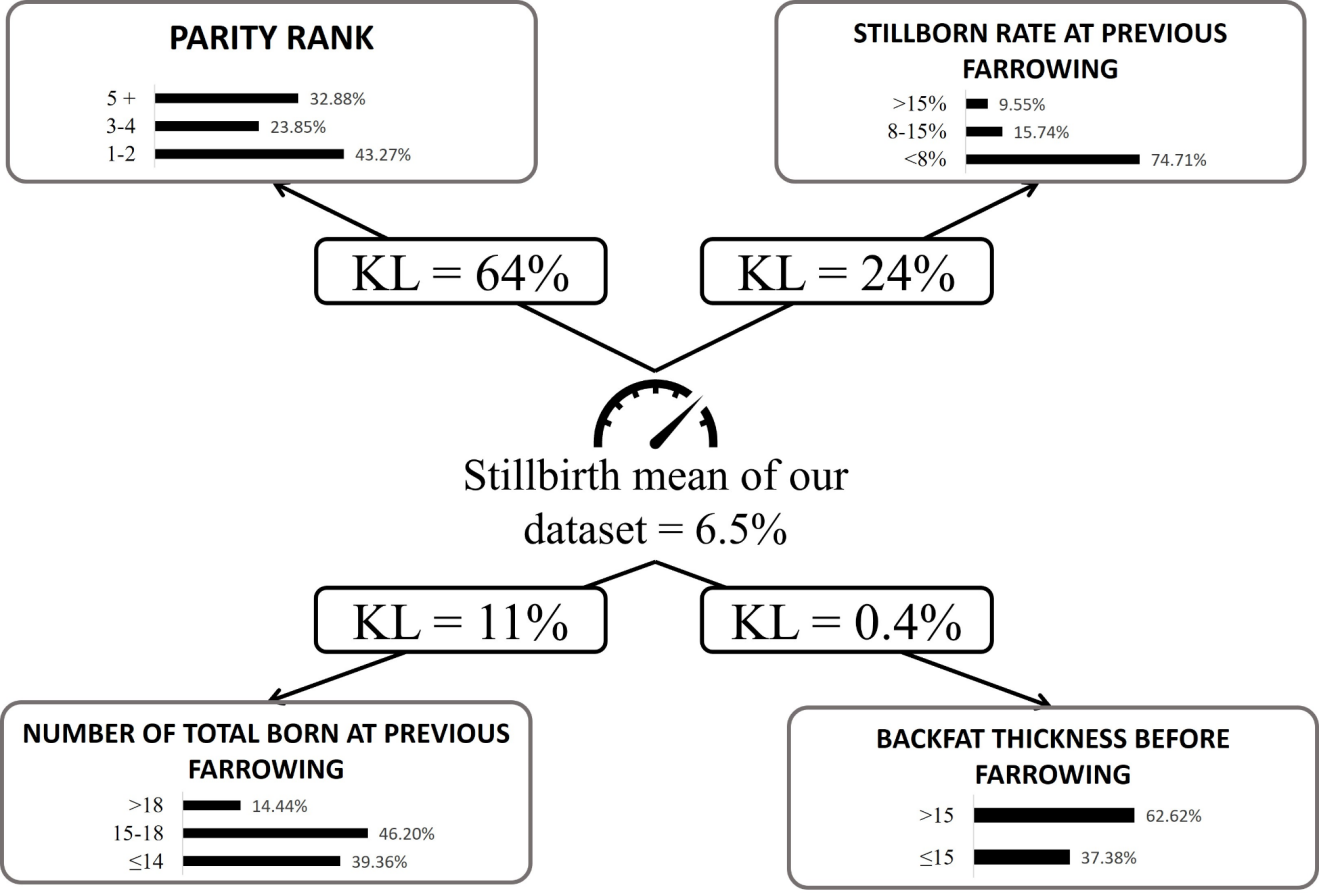
Final model obtained with BayesiaLab^®^ software KL: Kullback-Leibler divergences. The black bars represent the distribution for each variable.

The final model included nodes representing the variables and arcs (represented by arrows) representing the direct probabilistic relationships between the connected variables (Fig. 2). Each node is associated with a probability table describing the marginal probability distribution of the corresponding variable.

The strength of the relationships between two nodes directly connected by an arc is expressed by Kullback-Leibler (KL) divergences (i.e., percentages of mutual information). KL divergences for parity rank, %S_*n*−1_ and TB_*n*−1_ were 64%, 24% and 11%, respectively. This means that, for example, 11% of information (i.e. prediction) is explained by the number of total born at previous farrowing. Finally, the percentage of mutual information for the BFT variable is 0.4% if we consider all the parities. The characteristics of each significant variable are presented in the table below. For BFT before farrowing, this factor was significant only for parities 5 or more (p-value < 0.007). This is an important information to retain given the impact of body condition on farrowing performance.

**Table 4:** Overall analysis results

Nodes	Mutual information^1^	Normalized mutual information^2^	Prior mean value^3^	G-test^4^	Df^5^	*p*-value
Parity	0.0582	3.6749%	0.8961	297.6	4	**< 0.001**
%S_*n*−1_	0.0228	1.4416%	0.0562	116.8	4	**< 0.001**
TB_*n*−1_	0.0101	0.6384%	15.24	51.71	4	**< 0.001**
BFT	0.0003	0.0213%	16.66	1.725	2	0.388

^1^The mutual information measures the amount of information gained on variable “stillbirth (%S_n_)” by observing “Nodes”. ^2^Based on the mutual information, normalized mutual information includes a normalization factor. ^3^The prior mean value is the prior probability of event “stillbirth (%S_n_)”. ^4^The G-test is a type of independence test used to determine if there is a significant association between two categorical variables (for example, for the first line, the association between parity and stillbirth rate). ^5^Df represents the “degree of freedom” between each driver node and the target node (%Sn). Significant main effects are indicated in **bold** (p-value ≤ 0.05).

With this model, we were able to easily calculate risk percentages for different situations according to risk factor categories (Table 5). Deviations from the average are obtained according to the data used to create the model.

**Table 5:** Predicted stillborn rates and deviations from the average obtained from our dataset (6.5%) according to sow parity rank, stillbirth and prolificacy at previous farrowing and backfat thickness before farrowing

Parity rank	Percentage of stillborn at previous farrowing	Number of total born at previous farrowing	Predicted stillborn rate by the model	Deviations from average
Gilts and parity 2	S_*n*−1_<8%	TB_*n*−1_<15	3.6%	-45%
		15 ≤ TB_*n*−1_≤18	4.0%	-38%
		TB_*n*−1_>18	4.9%	-25%
	8%≤S_*n*−1_≤15%	TB_*n*−1_<15	4.7%	-28%
		15 ≤ TB_*n*−1_≤18	5.4%	-27%
		TB_*n*−1_>18	6.7%	3%
	S_*n*−1_>15%	TB_*n*−1_<15	5.4%	-17%
		15 ≤ TB_*n*−1_≤18	6.9%	6%
		TB_*n*−1_>18	8.5%	31%
Parities 3 and 4	S_*n*−1_<8%	TB_*n*−1_<15	4.9%	-25%
		15 ≤ TB_*n*−1_≤18	5.7%	-12%
		TB_*n*−1_>18	7.1%	9%
	8%≤S_*n*−1_≤15%	TB_*n*−1_<15	6.7%	3%
		15 ≤ TB_*n*−1_≤18	7.8%	20%
		TB_*n*−1_>18	9.5%	46%
	S_*n*−1_>15%	TB_*n*−1_<15	8.4%	29%
		15 ≤ TB_*n*−1_≤18	9.8%	51%
		TB_*n*−1_>18	11.6%	78%
Parities 5 and more	S_*n*−1_<8%	TB_*n*−1_<15	7.2%*	11%
		15 ≤ TB_*n*−1_≤18	8.4%*	29%
		TB_*n*−1_>18	10.2%*	57%
	8%≤S_*n*−1_≤15%	TB_*n*−1_<15	9.9%**	52%
		15 ≤ TB_*n*−1_≤18	11.4%**	75%
		TB_*n*−1_>18	13.1%**	102%
	S_*n*−1_>15%	TB_*n*−1_<15	12.5%**	92%
		15 ≤ TB_*n*−1_≤18	14.1%**	117%
		TB_*n*−1_>18	15.7%**	142%
		*	+ 1% if BFT ≤ 15 mm	
		**	+ 2% if BFT ≤ 15 mm	

*TB*_*n*−1_: *number of total born at previous farrowing*,* S*_*n−1*_: *stillborn rates at previous farrowing*,* BFT: backfat thickness*

For example, in the best conditions (namely a young sow that gave birth to fewer than 15 piglets at its previous farrowing with less than 8% stillborn), the model predicted a stillborn rate of 3.5% which represented a stillborn rate almost half that compared to the average risk of our dataset. In contrast, in the worst scenario – namely an older sow (parity five or more) with a high prolificacy (more than 18 piglets) and a marked stillborn rate (higher than 15%) at the previous farrowing – the predicted risk should be multiplied by 2.5 from 6.5 to 15.7%. Finally, if the sow was less than 15 mm at farrowing, the risk would increase further from 15.7 to 17.7%.

## Discussion

In this study, a Bayesian network was used to create a model to predict stillborn percentages according to different risk factors namely: parity rank, stillborn rate and prolificacy at the previous farrowing, and backfat thickness. Our results show that stillborn rates increase with parity, stillbirth at previous farrowing and previous litter size. This is in accordance with the results published by Blackwell in 1987 which, to our knowledge, has not yet been updated [26].

Two main characteristics were chosen to describe the model’s performance. First, the average value of calibration was 92% which represents the probability distribution of the model obtained according to our dataset. Second, the model accuracy was 72% (standard deviation 2.09%). This result shows that we have fewer than three chances out of 10 of giving a wrong prediction. This approach is strongly accurate for zootechnical studies based on field data [31]. Moreover, the model requires few parameters to calculate a risk of stillbirth making this tool easy to use and easy to implement in an operating system. The precision could perhaps have been improved with additional data such as: genetics, feed, season, and management because of the multifactorial nature of stillborn rates. However, a more elaborate model would be difficult to implement routinely on farms even if technical management software was available.

In our study, the average stillborn rate was 6.5%. It is also important to note that this study was conducted in three well-managed farms with an experienced workforce and good and consistent sanitary conditions. This finding implies that the stillborn rate was in line with or even relatively low according to the latest French and European benchmarks, even if the distribution of the data was large [5, 32]. Thus, further studies to test the model on farms where different characteristics (other management practices, different genetic types, etc.) occur are needed and would be helpful to validate our results. Moreover, the applicability of this model to farms facing any major health issues requires further investigation.

Parity is a widely demonstrated risk factor, and the link between parity and duration of farrowing can probably explain a portion of these reports [33]. Furthermore, litter size has increased considerably in recent decades, subsequently increasing a higher stillborn rate with a greater number of total born [3, 34]. Our results are in accordance with the findings of Muro et al. (2022), who highlighted that the stillbirth rate increased with the increasing total number of piglets [18].

The percentage of stillborn at the previous farrowing was the second most important factor highlighted by our model. Two different thresholds were used to discretise this variable (8 and 15% of stillborn) which allows us to be even more precise. Indeed, to our knowledge, there is no reference in the literature about such thresholds. Vanderhaeghe et al. (2010) showed that having more than one stillborn piglet at the time of the previous farrowing had a statistically significant impact on the stillborn rate at the time of the next farrowing [21]. Independent of the model, these cut-off values can therefore be useful in daily practice to ensure accurate monitoring of a sow’s performance development.

To a lesser extent, our model describes an increase in stillborn percentages for aged thin sows. In a previous study, Gourley et al. (2020) reported an increase in the stillborn rate in sows with decreasing backfat depth before farrowing [35]. This finding has been repeatedly confirmed in other studies [12, 36, 37]. However, in our study, a significant link between backfat thickness and the stillborn rate was not demonstrated for all parities but only for parities five or more. Even if only 0.4% of the mutual information is explained by this variable, we kept this data in our final model due to the fact that it is an easy-to-collect information in farms.

Overall, our model provides a useful framework for assisting in decision-making to reduce stillborn rates on pig farms. Indeed, several studies have demonstrated that attending farrowing could reduce stillbirths [38, 39]. Particularly, farrowing induction allow a better supervision facilitating human presence when needed. Monteiro et al. (2022) highlighted the benefit of farrowing induction as a tool for better obstetric assistance during farrowing and showed that this induction can reduce the risk of stillbirth by 28% [40]. Moreover, human presence helps to provide adequate care for piglets in the first hours of life. For example, the piglet’s oral and nasal cavities can be suctioned to clear any mucus or other debris during farrowing, which could improve mortality rate at birth. Through simulation analysis as well as sensitivity analysis, we demonstrated that the Bayesian network model could assist decision makers in identifying management actions that have the greatest influence on the percentage of stillborn.

## Conclusion

Three main risk factors were significant when considering stillborn rates: parity rank, total born and stillborn at previous farrowing. The optimal model for predicting the risk of producing stillborn also included backfat thickness before farrowing especially for sows with five or more pregnancies. However, further studies are needed to confirm the findings obtained thus far. These promising results could allow farmers to classify sows according to their risk of stillbirth and manage them accordingly to reduce piglet mortality at farrowing.

## Data Availability

The datasets used and/or analysed during the current study are available from the corresponding author on reasonable request.
